# The implication of Sir2 in replicative aging and senescence in *Saccharomyces cerevisiae*

**DOI:** 10.18632/aging.100299

**Published:** 2011-03-13

**Authors:** Cheol Woong Ha, Won-Ki Huh

**Affiliations:** School of Biological Sciences, Research Center for Functional Cellulomics, and Institute of Microbiology, Seoul National University, Seoul 151-747, Republic of Korea

**Keywords:** Sir2, aging, senescence, longevity, ribosomal DNA, TOR, sumoylation

## Abstract

The target of rapamycin (TOR) pathway regulates cell growth and aging in various organisms. In Saccharomyces cerevisiae, silent information regulator 2 (Sir2) modulates cellular senescence. Moreover, Sir2 plays a crucial role in promoting ribosomal DNA (rDNA) stability and longevity under TOR inhibition. Here we review the implication of rDNA stabilizers in longevity, discuss how Sir2 stabilizes rDNA under TOR inhibition and speculate on the link between sumoylation and Sir2-related pro-aging pathways.

## INTRODUCTION

Aging and longevity have always been an important issue in biological sciences. Several reports have indicated that calorie restriction leads to lifespan extension from yeast to humans [1-3]. Longevity effect is also seen when nutrient-sensing signaling pathways such as the target of rapamycin (TOR) pathway and insulin/IGF pathway are inhibited [4-7], and the molecular mechanisms that regulate these nutrient-dependent pathways can substantially promote longevity in various organisms and mammalian cells [8-13]. The budding yeast *Saccharomyces cerevisiae* provides valuable information on understanding aging process and identifying the molecular mechanisms that regulate longevity in eukaryotic organisms. Two types of aging, replicative and chronological, have been described in *S. cerevisiae*. Replicative aging is an aging model of mitotically active cells in which the lifespan of a mother cell is measured by the number of daughter cells produced before death [14, 15]. Chronological aging is an aging model of post-mitotic cells in which lifespan is defined by the survival time of cells in a non-dividing state [16]. In *S. cerevisiae*, a well-established mechanism for regulating longevity is the alteration of ribosomal DNA (rDNA) stability by the recombination between rDNA repeats and the accumulation of extrachromosomal rDNA circles (ERCs) [17]. Recently, we confirmed that the inhibition of TOR pathway stabilizes rDNA locus by enhancing the association of silent information regulator 2 (Sir2) with rDNA, thereby leading to replicative lifespan extension in *S. cerevisiae* [18]*.* In this research perspective, we review the implication of rDNA stabilizers in longevity regulation, discuss how Sir2 stabilizes rDNA under TOR inhibition and speculate on the link between sumoylation and Sir2-related aging pathway.

### Sir2 and the epigenetic regulators of yeast rDNA locus

Sir2 proteins, or sirtuins, are a well-known, highly conserved, family of NAD^+^-dependent deacetylases that are involved in the regulation of lifespan from yeast to humans [19-22]. Many reports have shown that sirtuins are linked to multiple physiological processes including metabolic regulation, DNA repair, stress response, apoptosis, cell survival and longevity [23-31]. Sirtuins also mediate the increased spontaneous physical activity in flies on calorie restriction and regulate p53 function via deacetylation in human cells [32, 33]. In *S. cerevisiae*, Sir2 is a functional component of epigenetic complexes required for the establishment of silenced chromatin regions in rDNA locus, telomeres and silent mating-type loci, *HML* and *HMR* [34-38]. *S. cerevisiae* rDNA consists of a 9.1-kb unit that contains RNA polymerase (Pol) I-transcribed 35S ribosomal RNA (rRNA) and Pol III-transcribed 5S rRNA gene, separated by a non-transcribed spacer, and is repeated 100-200 times on chromosome XII [39]. Because of highly repetitive nature, rDNA array is intrinsically unstable and is an easy target for homologous recombination. A primary cause of aging in *S. cerevisiae* is known to be the homologous recombination between rDNA repeats, which leads to the formation of ERCs that accumulate to toxic levels in mother cells [17]. Sir2 promotes replicative lifespan by repressing the homologous recombination between rDNA repeats and the subsequent formation of ERCs [40].

A well-known epigenetic regulator of yeast rDNA locus is the regulator of nucleolar silencing and telophase exit (RENT) complex that is composed of Sir2, Net1 and Cdc14 [41]. Net1, the core subunit of this complex, localizes to rDNA, recruits Sir2 to rDNA and is required for transcriptional silencing at rDNA [41-43]. Interestingly, several other epigenetic regulatory proteins also seem to be linked to rDNA stability and longevity. Tof2 interacts with Net1 and Sir2, binds to rDNA and induces rDNA silencing [43]. Lrs4 and Csm1, two of three subunits of monopolin complex that co-orients sister chromatids during meiosis I, interact with Tof2, associate with rDNA, establish rDNA silencing and suppress unequal recombination at rDNA [43-45]. Heh1 and Nur1, chromosome linkage inner nuclear membrane proteins, are physically linked to Lrs4 and Csm1, tether rDNA to nuclear periphery and promote rDNA stability [46]. Yeast linker histone Hho1, histone acetyltransferase Ada2 and Esa1 are required for rDNA silencing [47-49]. Histone methyltransferase Set1 is required for rDNA silencing in a Sir2-independent manner [50, 51]. Chromatin remodeling proteins, such as Snf2, Fun30, Isw1 and Isw2, establish silencing at rDNA and maintain rDNA chromatin structure [52-56]. Collectively, these findings suggest that rDNA silencing factors, histone-modifying enzymes and chromatin remodeling factors promote rDNA stability and regulate ERC-mediated aging in yeast.

### Sir2 is part of a pathway mediating the longevity effect of TORC1 inhibition

TOR kinase is a nutrient-responsive phosphatidylinositol kinase-related protein kinase structurally and functionally conserved from yeast to humans and plays critical roles in cell growth in response to nutrient availability by regulating transcription, translation, ribosome biogenesis and autophagy [13, 57-59]. In yeast, TOR kinase exists in two functionally distinct multiprotein complexes, TOR complex1 (TORC1) and TOR complex2 (TORC2), each of which signals via a different set of effector pathways [60]. An immunosuppressive and anticancer drug rapamycin specifically inhibits TORC1 and leads to a rapid decrease in ribosome biogenesis by regulating the transcription of all three kinds of RNA Pols [61, 62]. In past decade, it has been reported that TORC1 signaling is deeply involved in eukaryotic cell aging and aging-related diseases. The inhibition of TORC1 signaling by rapamycin can delay aging and prolong lifespan in *S. cerevisiae, Caenorhabditis elegans* and *Drosophila melanogaster* [63-66]. Recent studies have shown that rapamycin can significantly increase lifespan also in genetically heterogeneous mice [67, 68].

How the inhibition of TORC1 signaling extends lifespan is poorly understood. Meanwhile, whether Sir2 functions in rDNA stability and lifespan extension in yeast during TORC1 inhibition has been a matter of debate. Kaeberlein *et al.* reported that the inhibition of TORC1 signaling increases lifespan but has no effect on Sir2 activity [65], whereas Medvedik *et al.* indicated that TORC1 inhibition activates Sir2 by increasing the expression of *PNC1* encoding a nicotinamidase that depletes cellular nicotinamide, a physiological inhibitor of Sir2, thereby suppressing the formation of ERCs and leading to the extension of replicative lifespan [66]. Corroborating the latter study, we found that the inhibition of TORC1 signaling increases the association of Sir2 with rDNA in Pnc1- and Net1-dependent manners, enhances the transcriptional silencing of Pol II-transcribed genes at rDNA and induces the deacetylation of histones at rDNA, thereby promoting rDNA stability and replicative lifespan extension [18]. These findings suggest that Sir2 contributes to lifespan extension by enhancing rDNA silencing and rDNA stability under TORC1 inhibition.

Recent studies have reported that Sir2 inhibits replicative senescence by additional non-rDNA mechanisms. Sir2 is required for the asymmetric inheritance of oxidatively damaged proteins during cytokinesis, resulting in an enhanced capacity to respond to oxidative stress in daughter cells [69-72]. In addition, an age-associated decrease in Sir2 protein abundance is accompanied by the increase in histone acetylation and the loss of histones at specific subtelomeric regions in replicatively old yeast cells, indicating that Sir2 regulates replicative longevity through the maintenance of telomeric chromatin [73]. Whether TORC1 signaling modulates these non-rDNA functions of Sir2 is not known yet. It will be interesting to check whether the Sir2-dependent asymmetric inheritance of oxidatively damaged proteins and the Sir2-dependent maintenance of telomeric chromatin are influenced by TORC1 signaling.

### Sumoylation: a potential mechanism for the regulation of Sir2 function in rDNA maintenance

Small ubiquitin-like modifier (SUMO) modification is an important posttranslational mechanism for regulating protein function, especially in the nucleus and nucleolus. Sumoylation is involved in various nuclear functions such as transcription, DNA repair and nuclear domain organization [74, 75]. In yeast, SUMO is prominently enriched in the nucleolus and affects rDNA segregation and maintenance [76, 77]. Topoisomerase Top1, which is sumoylated by Siz1 and Siz2, facilitates rDNA transcription and replication, is required for rDNA silencing and maintains rDNA integrity [77, 78]. SUMO ligase Mms21, a subunit of the Smc5/Smc6 complex, binds to rDNA and maintains rDNA stability [79, 80]. Moreover, cohesin and condensin subunits, which play important roles in rDNA stability and structures, are sumoylated by Mms21, and the association of these subunits with rDNA is regulated by Mms21 [77]. Additionally, sumoylation of Rad52 suppresses rDNA recombination and affects the efficiency of recombinational DNA repair [79, 81]. These findings raise an interesting possibility that sumoylation may contribute to maintaining stable rDNA structure and thus promoting longevity.

Recent studies have shown that sumoylation is involved in the regulation of Sir2 function in the maintenance of chromatin structure at rDNA. Esc2, a member of a conserved family of proteins that contain SUMO-like domains, interacts with Sir2 through a SUMO-binding motif and is required for the maintenance of silent chromatin structure at rDNA [82]. A SUMO-binding motif of Esc2 is necessary and sufficient for interacting with both Sir2 and SUMO, and is required for the function of Esc2 in transcriptional silencing. These results raise a possibility that Sir2 is a sumoylated protein and Esc2 binds to sumoylated Sir2 via its SUMO-binding motif. A previous study has reported that the human sirtuin SIRT1 is sumoylated and this modification increases its deacetylase activity [83]. Although the sumoylation of yeast Sir2 has not been established yet, it is plausible that the activity of Sir2 may be regulated by its sumoylation. If Sir2 is not sumoylated, it is presumable that some sumoylated proteins may mediate its interaction with other proteins such as Esc2 and regulate its activity.

## CONCLUSION

**Figure 1.**
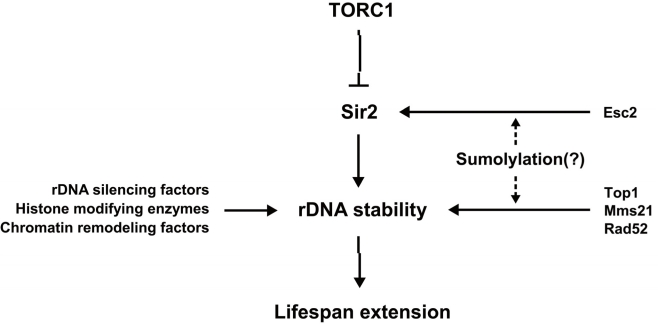
A model for the link between sumoylation and Sir2-related aging pathway Various factors stabilize rDNA to affect lifespan extension in *S. cerevisiae*. TORC1 inhibition, Sir2 and several epigenetic regulators of rDNA promote rDNA stability and longevity. Sumoylation may regulate the activity of Sir2 and contribute to maintaining stable rDNA structure, leading to lifespan extension.

Sirtuins are a highly conserved family of proteins that have been implicated in regulating diverse functions including longevity in a variety of species. Many studies suggest that Sir2 regulates rDNA recombination, DNA repair, chromosomal stability and longevity. In this research perspective, we briefly reviewed how Sir2 and other epigenetic regulators function as the stabilizer of rDNA and regulate longevity. Sir2 is part of a pathway mediating the longevity effect of TORC1 inhibition. Based on recent findings, we also propose that sumoylation may be involved in the regulation of Sir2 function in rDNA maintenance and thus longevity (Figure 1). Given that the human sirtuin SIRT1 is sumoylated and this modification increases its deacetylase activity [83], it will be interesting to investigate whether Sir2 and other sirtuins are sumoylated and, if so, whether their sumoylation is influenced by TORC1 signaling and how their functions are affected by sumoylation. These studies will provide deeper understanding of the mechanisms by which Sir2 and TORC1 signaling regulate longevity.
